# Compliance assessment of ambulatory Alzheimer patients to aid therapeutic decisions by healthcare professionals

**DOI:** 10.1186/1472-6963-10-232

**Published:** 2010-08-09

**Authors:** Oliver Schwalbe, Christian Scheerans, Ines Freiberg, Andrea Schmidt-Pokrzywniak, Andreas Stang, Charlotte Kloft

**Affiliations:** 1 Department of Clinical Pharmacy, Institute of Pharmacy, Martin-Luther-Universitaet Halle-Wittenberg, Wolfgang-Langenbeck-Str. 4, 06120 Halle, Germany; 2 Department of Clinical Pharmacy, Institute of Pharmacy, Freie Universitaet Berlin, Kelchstr. 31, 12169 Berlin, Germany; 3 Institute of Clinical Epidemiology, Medical Faculty of the Martin-Luther-Universitaet Halle-Wittenberg, Magdeburger Str. 8, 06097 Halle, Germany

## Abstract

**Background:**

Compliance represents a major determinant for the effectiveness of pharmacotherapy. Compliance reports summarising electronically compiled compliance data qualify healthcare needs and can be utilised as part of a compliance enhancing intervention. Nevertheless, evidence-based information on a sufficient level of compliance is scarce complicating the interpretation of compliance reports. The purpose of our pilot study was to determine the compliance of ambulatory Alzheimer patients to antidementia drugs under routine therapeutic use using electronic monitoring. In addition, the forgiveness of donepezil (i.e. its ability to sustain adequate pharmacological response despite suboptimal compliance) was characterised and evidence-based guidance for the interpretation of compliance reports was intended to be developed.

**Methods:**

We determined the compliance of four different antidementia drugs by electronic monitoring in 31 patients over six months. All patients were recruited from the gerontopsychiatric clinic of a university hospital as part of a pilot study. The so called medication event monitoring system (MEMS) was employed, consisting of a vial with a microprocessor in the lid which records the time (date, hour, minute) of every opening. Daily compliance served as primary outcome measure, defined as percentage of days with correctly administered doses of medication. In addition, pharmacokinetics and pharmacodynamics of donepezil were simulated to systematically assess therapeutic undersupply also incorporating study compliance patterns. Statistical analyses were performed with SPSS and Microsoft Excel.

**Results:**

Median daily compliance was 94% (range 48%-99%). Ten patients (32%) were non-compliant at least for one month. One-sixth of patients taking donepezil displayed periods of therapeutic undersupply. For 10 mg and 5 mg donepezil once-daily dosing, the estimated forgiveness of donepezil was 80% and 90% daily compliance or two and one dosage omissions at steady state, respectively. Based on the simulation findings we developed rules for the evidence-based interpretation of donepezil compliance reports.

**Conclusions:**

Compliance in ambulatory Alzheimer patients was for the first time assessed under routine conditions using electronic monitoring: On average compliance was relatively high but variable between patients. The approach of pharmacokinetic/pharmacodynamic *in silico *simulations was suitable to characterise the forgiveness of donepezil suggesting evidence-based recommendations for the interpretation of compliance reports.

## Background

Alzheimer's disease presents the most common type of dementia, accounting for 50%-60% of all cases [1]. In 2001, more than 24 million people worldwide were suffering from dementia, a number that is expected to double every 20 years up to 81 million in 2040 due to increase in life expectancy [1]. Cholinesterase inhibitors such as donepezil, galantamine and rivastigmine and the NMDA-receptor modulator memantine present the first-line pharmacotherapy for Alzheimer's disease [2,3]. Prescription data from a large statutory health insurance in Germany show that donepezil and memantine are the market leaders, each representing approximately one third of all defined daily doses of all four agents [4]. These agents can improve symptoms, primarily in the domains of cognition (e.g. ADAS-cog score) and global function [5]. Effectiveness should be evaluated in two to four months intervals [6].

Within outcomes research compliance presents a treatment modifier, which highly impacts the effectiveness of pharmacotherapy [7]. Studies investigating compliance show a variety of measures of medication usage and varying terminologies (e.g. compliance, adherence, persistence) complicating the interpretation and comparison of those studies [8,9]. The Medication Compliance and Persistence Work Group of the International Society of Pharmacoeconomics and Outcomes Research (ISPOR) defined medication compliance as "the extent to which a patient acts in accordance with the prescribed interval and dose of a dosing regimen"[8]. Persistence may be referred to as "the duration of time from initiation to discontinuation". Today, no overarching term combines these two concepts [8].

Since the end of the 1970s electronic monitoring has been used to compile dose administration histories of ambulatory patients [10]. The so called medication event monitoring system (MEMS) consists of a vial with a microprocessor in the lid which records the time (date, hour, minute) of every opening [11]. In contrast to traditional compliance assessment methods such as pill count, patient diaries or patient self-report, the method of electronic monitoring demonstrated to be a more reliable tool allowing a detailed analysis of patient medication taking behaviour over time [12]. However, as indirect method actual ingestion of the medicine cannot be measured [10] and compliance may be underestimated (e.g. in the case that a weekly pill-box is used instead) [13]. In addition, MEMS in itself presents a compliance enhancing intervention for patients who are informed about the purpose of the device. This may lead to an overestimation of their "usual" medication taking behaviour. Nevertheless, electronic monitoring has been recognised closest to a 'gold standard' for compliance measurement [11]. To our knowledge, compliance studies among Alzheimer patients using electronic monitoring have not been conducted until now. Previous studies have employed pharmacy refill data to investigate medication taking behaviour [14-17]. Reports summarising MEMS data characterise compliance-time patterns in detail and can be utilised as part of a compliance enhancing intervention where a healthcare professional provides feedback to the patient on his/her medication taking behaviour [18]. This was termed measurement-guided medication management (MGMM).

The advent of electronic monitoring has also advanced research on the question "how much compliance is enough?" being closely related to the concept of forgiveness. Urquhart defined forgiveness as the "drug's post-dose duration of action minus the prescribed interval between doses" [10]. Researchers in the HIV area have adopted a more general definition of forgiveness as ability of a regimen to achieve and sustain adequate pharmacological response (in this case viral suppression) despite suboptimal compliance [19]. In the present work forgiveness is used in the latter sense, specifying the former as forgiveness according to Urquhart. The crucial 'experiment' for measuring how much compliance is sufficient, presents the controlled, blinded substitution of placebos for active drug [20]. This is frequently not ethically possible and has only been pursued in the field of e.g. oral contraception, hypertension and depression [20,21]. Furthermore, a correlation between compliance and clinical outcome was established in observational studies [22,23].

The capacity for forgiveness of drugs may differ substantially, depending on their pharmacokinetic (PK, i.e. the drug exposition) and pharmacodynamic (PD, i.e. the drug effect) properties, e.g. clearance and steepness of concentration-effect relationship. Thus, given a known pharmacokinetic/pharmacodynamic (PK/PD) relationship, *in silico *studies were also suggested for the characterisation of forgiveness [24,25]. In the case of donepezil, the degree of inhibition of peripheral cholinesterase has been identified as a PD biomarker [26]. In this work we defined "time with therapeutic undersupply" (TTU) as a parameter. To avoid TTU, the daily dosages of cholinesterase inhibitors to achieve a consistent red blood cell (RBC) cholinesterase inhibition of at least 40% corresponded to those causing improvements in ADAS-cog and functional activity scores [27].

The aim of our pilot study was to determine the compliance of ambulatory Alzheimer patients to antidementia drugs under routine therapeutic use by means of electronic monitoring over a six months period. In addition, we performed pharmacokinetic/pharmacodynamic *in silico *simulations using the pilot study compliance pattern data and published PK/PD models to characterise the forgiveness of donepezil, the most frequently prescribed antidementia drug. Ultimately, we aimed at developing a recommendation for the evidence-based interpretation of donepezil compliance reports which may form the basis of a MGMM intervention.

## Methods

### Study design

The current research was part of a sequential phase II trial (pilot study) within the Medical Research Council framework [28] (guidance for the development and evaluation of complex interventions) investigating the impact of a pharmaceutical care intervention on ambulatory Alzheimer patients' compliance and other outcomes such as knowledge in pharmacotherapy and caregivers' health-related quality of life. Our pharmaceutical care program in the second part of the trial should include a complex intervention including MGMM. The study was approved by the respective ethics committee (Ethics Board 4) of the Charité - Universitaetsmedizin Berlin, Germany. Patients had to fulfil the following inclusion criteria: diagnosis of Alzheimer's disease; prescription of an antidementia drug (ATC-code N06D); living in ambulatory setting; sufficient command of the German language (patient and caregiver); ability to consent (patient). Patients who lived in residential homes or who intended to move further away during the study period were not eligible according to study protocol. Patients were consecutively recruited from the above mentioned clinic. The study design comprised a one month run-in phase followed by a main phase lasting six months. All study individuals screened between March 2006 and July 2007 were Alzheimer patients of the gerontopsychiatric clinic of the Charité - Universitaetsmedizin Berlin, Germany. The recruited patients were a convenience sample and comprised the standard care group of the phase II trial.

Standard care denoted community pharmacists provided their usual dispensing service that included appropriate drug information and advice for patients according to the Ordinance on the Operation of Pharmacies [29]. No defined compliance enhancing intervention to the patient or caregiver was offered. Having obtained written informed consent from patients and caregivers, the antidementia drug was repacked into a MEMS container. Patients and caregivers were informed about the monitoring device. Each patient/caregiver was given the instruction to open the MEMS container when they wanted to take a medicine, to remove and take the prescribed dose, then promptly close the device. Additionally, patients' regular community pharmacy was informed and trained how to perform and document the repackaging procedure.

### Compliance

Compliance was determined using MEMS 6 TrackCap (Aardex Ltd., Zug, Switzerland). Refills of MEMS vials were performed and documented in patients' regular community pharmacies. Subsequently, refill events and self-reported non-usage periods (e.g. due to hospital stays) were removed from the dataset. Daily compliance in the main phase served as primary outcome measure, defined as percentage of days with the correct number of MEMS openings (e.g. two openings for a twice daily drug) acting on the usual assumption that opening corresponded to drug intake. Daily compliance was calculated for each individual month as well as the total main phase comprising six months. Additionally patients were dichotomised into 'compliers' (daily compliance ≥ 80% according to the commonly employed cut-off criterion) and 'non-compliers' (daily compliance < 80%) [30]. For descriptive data analysis of MEMS recordings of our study, means or medians were calculated as measure of central tendency. According to the attributes of the respective variables, we calculated range, quartiles and 95% confidence interval. In contrast to the large amount of MEMS data the sample size of patients was small. In this part, no statistical test or extrapolations were performed based on individual data. Statistical analyses were performed with SPSS 15 (SPSS Inc., Chicago, Ill, USA).

### Forgiveness

To characterise the forgiveness of donepezil, PK/PD *in silico *simulations were performed using Microsoft Excel 2003 (Microsoft Corp., Redmond, WA, USA). Three approaches (A, B and C) were applied via trace-driven simulations [31] using different compliance patterns as input (function): Approach A used the compliance data from the pilot study to evaluate therapeutic undersupply for all individual patients taking donepezil. Approaches B and C served as sensitivity analyses to characterise the forgiveness of donepezil. For approach B, discrete daily compliance values (0%-100%) were simulated using a step size of 10%. These selected compliance patterns were created by the pseudo-random number generator in Microsoft Excel, for a period of 200 days. Eventually for approach C, scenarios of 1-7 dosage omissions at steady-state after a 14 days run-in phase and with a 7 days follow-up phase were simulated. The utilised PK and PD models are summarised in Table 1.

**Table 1:** Pharmacokinetic and pharmacodynamic models for donepezil.

Model type (name) & equation	Variables and parameters		Reference
*Pharmacokinetic model * (two compartment model)			
$C=D\cdot \left[\begin{array}{l} A\cdot e^{-\alpha (t-lag\;time)}+\\ B\cdot e^{-\beta (t-lag\;time)-}\\ (A+B)\cdot e^{-k_{a}\cdot (t-lag\;time)} \end{array}\right]$	for D = 5 mg: A = 3.502 ng/mL B = 1.209 ng/mL α = 0.445 h^-1^ β = 0.014 h k_a _= 1.319 h^-1^ lag time = 0.96 h	for D = 10 mg: A = 4.536 ng/mL B = 1.234 ng/mL α = 0.542 h^-1^ β = 0.015 h^-1^ k_a _= 1.696 h^-1^ lag time = 0.68 h	[32]
*Pharmacodynamic model * (E_max _model)	E_max _= 100.8% EC_50 _= 15.6 ng/mL		[34]
$E=\frac{E_{\max}\cdot C}{EC_{50}+C}$			

Parameter values and reference to literature, utilised for the *in silico *simulation.

C = total plasma drug concentration [ng/mL]

t = time [h]

D = dose

A, B = intercepts of the two exponential terms

α, β = macro (hybrid) rate constants

k_a _= absorption rate constant

E = effect, i.e. inhibition of peripheral cholinesterase, %

E_max _= maximum effect

EC_50 _= drug concentration at half of maximum effect

For donepezil, linear PK was assumed [27].The plasma concentration-time course of donepezil for long-term treatment with (irregular) multiple dosing (up to 200 days with Δt = 1 min) was generated using a previously published PK model [32], applying the principle of superposition, as explained in [33]. In case of approach C where dosage omissions at steady state conditions were analysed, the dosing interval between the last and next dose administered was accordingly extended, e.g. doubled (48 h instead of 24 h) for one missing dose.

The resulting plasma concentration-time course of donepezil (for multiple dosing) was directly linked to the PD model (see Table 1) describing the inhibition of peripheral cholinesterase that served as PD biomarker [34]. The main outcome variable for all three simulations was the "time with therapeutic undersupply" (TTU). TTU was defined as time (percentage or hour) with the PD biomarker below the minimum therapeutic inhibition, that is < 40% inhibition of peripheral cholinesterase. Additionally, for approach C forgiveness according to Urquhart was determined as the "drug's post-dose duration of action minus the prescribed interval between doses" [10].

## Results

### Compliance

From 39 patients who fulfilled the inclusion criteria 31 (79%) were enrolled into the study. The main reason (6 out of 8 refusals) for nonparticipation was MEMS-related inconveniences (e.g. incompatibility of MEMS and use of weekly pill-boxes).

Fifty percent of all patients were between 71 and 82 years old and both sexes were almost equally represented. Duration of antidementia pharmacotherapy at inclusion ranged from drug-naïve to 6.5 years with 2-12 concomitantly administered drugs. The majority of patients were on a once daily antidementia regimen with donepezil being the most prevailing drug. Moreover, in more than two-thirds of all patients the caregiver only was responsible for pharmacotherapy (Table 2). In total, 6507 MEMS openings were recorded over 5399 days.

**Table 2:** Characteristics of the patients at baseline (n = 31) and study characteristics.

Characteristics	
	*n (%)*
Sex: women	17 (55)
Responsibility for pharmacotherapy	
Patient only	2 (6)
Patient supported by caregiver	6 (19)
Caregiver only	22 (71)
Professional care only	1 (3)
Antidementia drugs in MEMS^a^	
Donepezil	12 (39)
Galantamine	12 (39)
Memantine	7 (23)
Rivastigmine	1 (3)
Regimen of antidementia drug in MEMS^a^	
Once daily	24 (75)
Twice daily	8 (25)
	
	*median (range)*
Age in years	76 (47-96)
Duration of MEMS monitoring in days^b^	180 (140-180)
Duration of antidementia pharmacotherapy at inclusion in months	18 (0-78)
Number of regularly administered drugs	6 (2-12)

MEMS, medication event monitoring system; ^a ^one patient was started on donepezil (MEMS monitoring during 49 days), but later in the course of the study he was switched to memantine (MEMS monitoring during 111 days); ^b ^eight out of 31 patients (26%) had non-monitored periods (e.g. due to hospital stays) or incomplete follow-up of less than 180 days.

**Figure 1:**
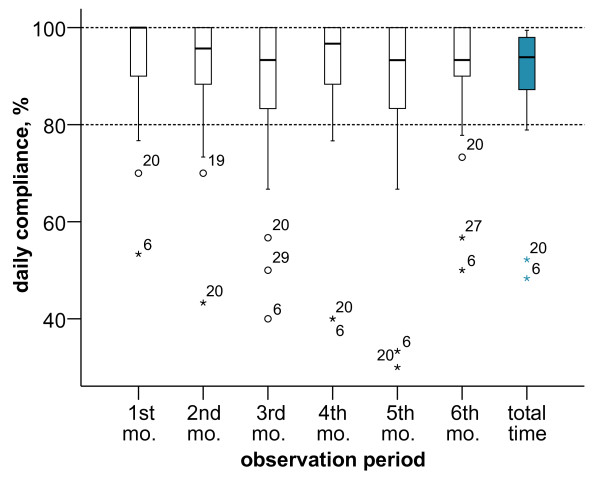
**Daily compliance during study observation period for each month and the entire observation period**. The ends of the whiskers represent the lowest data value within 1.5 times the box height from the lower box edge and the highest data value within 1.5 times the box height from the upper box edge, respectively. Index numbers represent individual patients ID: if associated with circle, this ID was regarded as outlier (1.5-3 times box height from the box edge), if with a star as extreme value (> 3 times box height from the box edge); mo. = month.

Median compliance of all patients in the main phase was 94% with a large range of 48% to 99% (lower quartile: 87%, upper quartile: 98%). In comparison to the first month, median compliance decreased by 7% points in the sixth month (Fig. 1).

**Figure 2:**
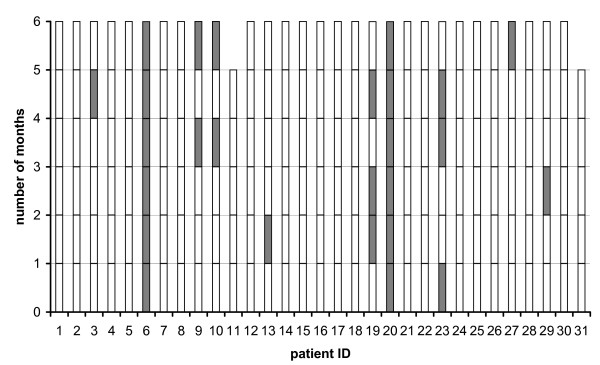
**Intraindividual daily compliance during the observation period**. Compliant months (daily compliance ≥ 80%, white bars) or non-compliant months (daily compliance < 80%, grey bars); patient #11 and patient #31 participated five months only.

After dichotomisation of compliance (daily compliance 80% and more or < 80%), intraindividual compliance patterns by month revealed that 10 patients (32%, 95% confidence interval: 17%-51%) were at least one month non-compliant (Fig. 2). Among these, two patients were non-compliant throughout the main phase.

### Forgiveness of donepezil

To determine their possible therapeutic undersupply individual compliance patterns of the twelve patients of the study comprising 1873 MEMS recordings with either 5 mg (n = 3) or 10 mg (n = 9) once daily donepezil were simulated (approach A). In the 5 mg donepezil dosing group, two patients (#13 and #23) out of three were undersupplied during certain time periods. Moreover, for both patients it was found that one occasionally omitted dose (dosing interval ≈48 h) did not cause therapeutic undersupply. However, series of one dose omission triggered the appearance of undersupplied periods. Very interestingly, daily compliance in the main phase for patient #13 and #23 was 87.2% and 80.0%, respectively, i.e. they were regarded as compliant according to the commonly employed 80% cut-off criterion. None of the patients taking 10 mg donepezil displayed any undersupplied periods.

**Figure 3:**
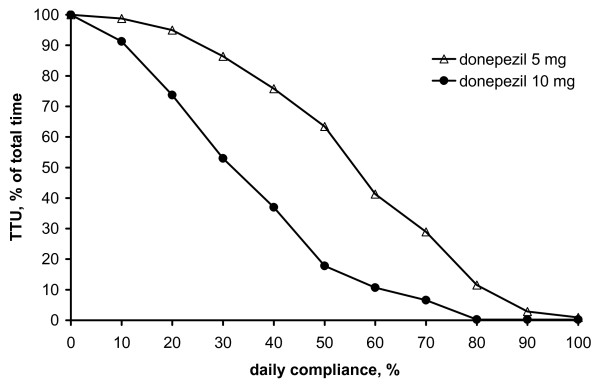
**Forgiveness characterisation: Simulation approach B**. Time with therapeutic undersupply (TTU) in percentage of total time versus discrete daily compliance values.

Simulations of discrete compliance values are displayed in Fig. 3 (approach B). For 10 mg (5 mg) donepezil negligible therapeutic undersupply was observed with daily compliance exceeding 80% (90%) leading to 0.3% (2.9%) TTU of total time. Fig. 3 revealed a sigmoidal relationship between TTU and discrete compliance values. For efficacious pharmacotherapy, however, TTU of total time should be negligible, i.e. close to zero.

**Figure 4:**
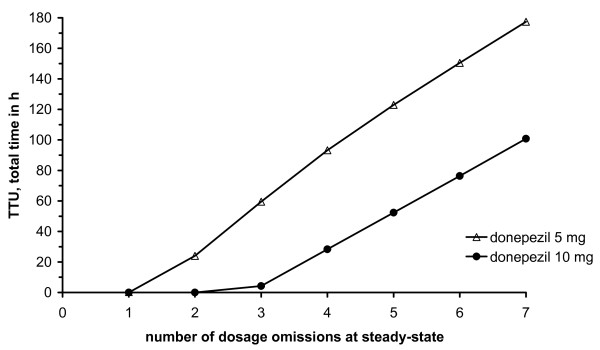
**Forgiveness characterisation: Simulation approach C**. Total time with therapeutic undersupply (TTU) in hours versus number of dosage omissions at steady-state.

Approach C characterised the influence of 1-7 dosage omissions on the inhibition of cholinesterase (Fig. 4): Initially, both curves ran parallel to the abscissa. Subsequently, TTU linearly increased with a higher number of dosage omissions with comparable slope. In the case of 5 mg donepezil two or more dosage omissions caused therapeutic undersupply. For 10 mg three or more dosage omissions at steady state led to therapeutic undersupply. Forgiveness according to Urquhart was calculated as 68.4 h for 10 mg and 28.3 h for 5 mg donepezil, respectively, i.e. the PD effect lasted for almost 3 d and more than 1 d beyond the dosing interval, respectively.

### Evidence-based interpretation of compliance reports for donepezil

Based on our results, for 5 mg donepezil, a monthly daily compliance less than 90% or two consecutive dosage omissions were regarded as therapeutically relevant non-compliance. For 10 mg donepezil we regarded a monthly daily compliance of less than 80% or three consecutive dosage omissions as a trigger of therapeutically relevant non-compliance.

## Discussion

In this study, we provide evidence from non-invasively acquired data that compliance of ambulatory Alzheimer patients in their daily life seems to be relatively high with on average one non-compliant day out of ten. Nevertheless, about one third of all patients were at least one out of six months non-compliant employing the frequently used 80% cut-off criterion. PK/PD *in silico *simulations revealed that for 10 mg (5 mg) donepezil forgiveness of donepezil was estimated as 80% (90%) daily compliance or *two *(*one*) dosage omissions at steady-state, respectively. Based on these results, evidence-based recommendations for the detection of relevant non-compliance were developed to aid the interpretation of compliance reports as part of e.g. a MGMM.

Electronic monitoring of medication events - based on electronic detection of opening a container - presents an indirect method of estimating when and how much drug has been taken. Direct measures such as directly observed therapy and therapeutic drug monitoring may be feasible in certain areas (e.g. intensive care units) but not in others, as in our setting and patient population due to the high effort required. Errors in compliance estimation will occur if a patient opens the container without taking the drug or takes another number of tablets than the one prescribed. In addition, apparently irrational openings could also be detected in the investigated population (in 61% of all patients, but only in approximately 1% of all openings). Arnet and Haefeli recognised so called "curiosity events" in nearly 20% of patients' electronic monitoring systems which were due to showing the device to relatives or friends or uncertainty whether they had closed the electronic monitoring device [35].

Denhaerynck et al. provided a form to their patients to document deviations from "normal" usage of the monitoring device (e.g. during hospital stays) as well as conducted a structured interview with each patient at the end of the study [36]: Uncensored data showed a 3.4% higher non-compliance than the censored data (incorporating information of forms and interviews). Hence, non-censoring could be considered as an overestimation of non-compliance [36]. Nevertheless, another study revealed that uncorrected electronic monitoring data could successfully project measured plasma concentrations of drugs [37]. In our study, pharmacy refills and self-reported non-usage periods (e.g. due to hospital stays) were excluded from further analysis which may relevantly improve the quality of the data.

Denhaerynck et al. investigated the compliance enhancing effect in transplant patients by omitting the first month of electronic monitoring from their analysis: This only resulted in a 0.4% decrease in daily compliance. They concluded that the compliance enhancing effect of electronic monitoring only had minor clinical significance [36]. Nevertheless, the run-in phase of one month as part of our study design may reduce this effect, thus giving a more realistic picture of medication taking behaviour.

Electronic monitoring presents the most reliable method for the determination of compliance despite certain disadvantages [11]. Three studies determined persistence for donepezil and rivastigmine in treatment naïve patients using pharmacy-refill data [14-17]. Nearly one third of all patients stopped donepezil or rivastigmine therapy within 60 days of starting therapy [15]. There were no marked differences in persistence between both drugs [15,17]. Especially during the dosage up-titration period, adverse drug reactions (mainly gastrointestinal) occur that usually cease later in the course [38]. With the exception of one patient there were no treatment naïve participants in our study being particularly prone to adverse drug reactions. This patient was down-titrated to 5 mg donepezil from 10 mg due to adverse drug effects but did not stop therapy. In the study conducted by Roe and co-workers 14% of all patients who continued therapy for at least 6 months showed gaps in supply for at least six weeks [16]. In our study, around one third of all patients were considered non-compliant (80% cut-off criterion) in at least one month demonstrating how much more sensitive electronic monitoring has to be regarded compared to pharmacy refill data. During the course of the study, median compliance slightly decreased by 7% points. In future, a longer study should be performed to assess whether the decrease will be more substantial over a longer period.

Inhibition of peripheral cholinesterase served as a biomarker of pharmacological response in Alzheimer's disease. Jann et al. concluded that daily doses of cholinesterase inhibitors to achieve a consistent peripheral cholinesterase inhibition of at least 40% corresponded to those leading to an improvement in cognition and functional activity scores [27]. Elsewhere, positive associations were reported between the AChE inhibition and change in ADAS-cog and CIBIC plus [34]. Unfortunately, the strength of association was not reported by Rogers et al. and thus the predictive value of RBC AChE inhibition has been discussed [34,39] it nevertheless provides evidence of potential efficacy. Hence, 40% inhibition served as a reasonable cut-off below which only minor efficacy has to be expected.

Donepezil was chosen for characterising the forgiveness in the *in silico *analysis due to the following reasons: (i) most widely used acetylcholinesterase inhibitor, (ii) high number of MEMS data available (2720 monitored days), and (iii) an available PK as well as PD model and an established dose-response relationship.

The PK model included the long half-life of donepezil of 50 h. Since population pharmacokinetic models that provide variability parameters are not available, PK variability could not be employed in the *in silico *study. Moreover, for approaches B and C, investigating the influence of daily compliance and dosage omission on therapeutic coverage/undersupply, a high magnitude of PK variability could provide a more sophisticated picture of the outcome. Future *in silico *simulations for donepezil should account for population PK models that quantify variability, when available. In addition, subgroup analysis for confounders should be performed with a larger data base considering the metabolising isoenzymes, cytochrome P450 3A4 and the 2D6 which can be induced or inhibited by comedication. Besides, the current simulation had to be limited to donepezil because for other antidementia drugs literature data was mostly insufficient but highly warranted. For galantamine, a PK model was available but no PD model [40]. An exception presented rivastigmine as oral capsules where a PK/PD model was described in the literature [41]. Due to the low number of rivastigmine data of our study (one patient only), its low market share (13%) [4] and increasing acceptance of a transdermal formulation with differing PK/PD characteristics lacking published PK models we did not find it adequate to perform a simulation.

From three patients being prescribed donepezil who were at least one month non-compliant (daily compliance < 80%) two displayed periods of therapeutic undersupply. Especially the combination of a drug holiday (dosing interval exceeding 96 h) and several single dose omissions triggered therapeutic undersupply. The long half-life of donepezil (~50 h) might at first glance exclude this drug from being interesting for forgiveness analysis. But as shown in our investigation, forgiveness is in fact an issue for donepezil in certain non-compliance patterns (e.g. several dose omissions). These patterns in electronic monitoring reports present an indicator of potential insufficient therapeutic coverage and have to be discussed with the patient/caregiver [18]. In the absence of electronic monitoring patients or caregivers should be questioned in an empathic, non-patriarchic style about their medication taking behaviour. An old, still widely-held idea going back to research in the cardiovascular field in the 1960 s, was that taking 80% of the prescribed doses generally qualifies as satisfactory compliance [42]. This view, however, has to be regarded as pharmacodynamically naïve since forgiveness of each drug product is determined by its individual dosage form, PK and PD [43]. Our results reveal that 80% compliance already results in more than 10% therapeutic undersupply for 5 mg donepezil daily whereas 10 mg daily maintains almost full therapeutic coverage, i.e. for 5 mg donepezil we now suggest 90% compliance to serve as a cut-off.

Both donepezil dosages forgive a common compliance error: one occasionally omitted tablet. Compared to other therapeutic areas donepezil exhibited a high degree of forgiveness. If a single gestagen-only pill is taken more than three hours late, there will be a need of back-up contraception [44]. The forgiveness of once daily antihypertensives atenolol and betaxolol can be estimated as about 6 hours and more than 48 hours [45]. These results were generated by controlled (verum only group), blinded trials partially substituting verum against placebo, whereas we have implemented *in silico *simulations, in this way providing a lower level of evidence. Studies early in the era of antiretroviral therapy demonstrated the need for > 95% compliance in order to achieve and sustain viral suppression [19]. High rates of viral suppression could also be attained at more moderate compliance with newer antiretroviral regimens (e.g. lopinavir/ritonavir) [23]. Beyond this, sparse or no evidence is available on forgiveness of major therapeutic classes.

## Conclusions

For the first time, this pilot study assessed compliance under routine therapeutic use of ambulatory Alzheimer patients using electronic monitoring. Compliance in ambulatory Alzheimer patients was relatively high but variable. The approach of pharmacokinetic/pharmacodynamic *in silico *simulations was suitable to characterise the forgiveness of donepezil giving evidence-based recommendations for the design of the intervention part of our study (MGMM-guided complex intervention of the pharmaceutical care program) and for the interpretation of compliance reports. Information on patients' compliance (percentage *and *pattern) should be incorporated in decisions whether to continue therapy in the case of therapeutic failure.

## Competing interests

The authors declare that they have no competing interests.

## Authors' contributions

OS (having full access to all the data in the study) and CK designed the study, acquired, analyzed and interpreted the data, responsibility for the integrity of the data and the accuracy of the data analysis and drafted the manuscript. AS and ASP were involved in the study concept and design, analysis and interpretation of the data and the critical revision of the manuscript for important intellectual content. CS was involved in the design, analysis, and interpretation of the simulation study. IF was involved in the analysis and interpretation of the compliance data. All authors read and approved the final manuscript.

## Pre-publication history

The pre-publication history for this paper can be accessed here:

http://www.biomedcentral.com/1472-6963/10/232/prepub
